# Platelet-rich fibrin and chitosan dressings for healing after third molar extractions

**DOI:** 10.6026/973206300210858

**Published:** 2025-04-30

**Authors:** Ramakrishna Maiya G.R, Gururaju C.R, Siri Chandana Gonabhavi, Sunil Kumar Biraggari, Deepalakshmi S., Vinay Rao

**Affiliations:** 1 Department of Oral and Maxillofacial Surgery, Sharavathi Dental College and Hospital, Shimoga, Karnataka, India; 2 Department of Periodontics and Implantology, G. Pulla Reddy Dental College and Hospital, Kurnool, Andhra Pradesh, India; 3 Department of Oral Medicine and Radiology, Dr. D.Y. Patil School of Dentistry, Nerul, Navi Mumbai, Maharashtra, India; 4 Department of Conservative Dentistry, Endodontics and Aesthetic Dentistry, AMC Dental College, Ahmedabad, Gujarat, India

**Keywords:** Platelet-rich fibrin, chitosan-based dressing, mandibular third molar extraction, postoperative healing, randomized clinical trial

## Abstract

The surgical removal of lower wisdom teeth produces both postoperative discomfort and treatment healing delays. Therefore, it is of interest to evaluate PRF therapy alongside chitosan-based dressing as treatment for 60 bilateral extraction patients. The patients who received PRF therapy experienced less pain (VAS score 3.5 vs 4.2) together with less swelling (70% reduction compared to 50%) and better healing scores (9.2 vs 8.1). The evaluative measurements using VAS and Landry's scale occurred on days 3 and 7 and day 14. The recommended treatment for boosted postoperative recovery reveals PRF as delivering better healing results than others.

## Background:

The extraction of mandibular third molars, commonly referred to as wisdom teeth, is a routine oral surgical procedure performed worldwide. This surgery is often indicated for reasons such as impaction, pericoronitis, crowding, or pathology associated with the third molars. Platelet-rich biomaterials and advanced hemostatic agents have shown promising results in controlling post-extraction bleeding in patients on antiplatelet therapy. The use of biocompatible hemostatic materials is increasingly supported in dental practice for enhancing wound healing and minimizing complications in medically compromised patients [1, 2]. Managing these postoperative complications effectively is critical to improving patient outcomes and ensuring a faster return to normalcy. Various pharmacological and non-pharmacological interventions have been explored to enhance recovery, including the use of advanced wound care materials. Among these, platelet-rich fibrin (PRF) and chitosan-based dressings have garnered significant attention for their potential to improve healing in surgical wounds.

Platelet-rich fibrin (PRF) is an autologous biomaterial derived from the patient's own blood. It is classified as a second-generation platelet concentrate and is known for its high concentration of growth factors such as vascular endothelial growth factor (VEGF), platelet-derived growth factor (PDGF) and transforming growth factor-beta (TGF-β). These growth factors play a pivotal role in tissue regeneration by enhancing angiogenesis, stimulating fibroblast proliferation and promoting epithelialization and collagen deposition at the wound site [3, 4]. In addition to its regenerative properties, PRF serves as a biological scaffold, creating an optimal environment for cell migration and proliferation, which accelerates the healing process. On the other hand, chitosan-based dressings are derived from chitin, a natural polysaccharide found in the exoskeleton of crustaceans. Chitosan is widely recognized for its biocompatibility, biodegradability and antimicrobial properties. These dressings maintain a moist environment at the wound site, which is essential for optimal healing and they have been shown to prevent microbial infections effectively. Moreover, chitosan has hemostatic properties, aiding in the control of bleeding at surgical sites and can act as a barrier to external contaminants [5, 6. Its use in various surgical fields, including dermatology and orthopedics, has demonstrated promising results in reducing wound closure time and minimizing complications [7]. Although both PRF and chitosan-based dressings have shown individual efficacy in promoting wound healing, direct comparisons of their effectiveness in oral surgery, particularly in mandibular third molar extraction sites, remain scarce. The need for such comparative studies is critical to guiding clinicians in selecting the most effective modality for postoperative care. Understanding the relative benefits and limitations of these materials could help optimize healing, reduce the burden of complications and improve overall patient satisfaction. Therefore, it is of ineterst to address this gap by comparing postoperative healing outcomes using PRF and chitosan-based dressings in mandibular third molar extractions. The parameters evaluated include postoperative pain, swelling and wound healing progression. By providing evidence-based insights, this study seeks to determine the superior modality for enhancing recovery and improving clinical outcomes in patients undergoing this routine yet challenging oral surgical procedure.

## Materials and Methods:

## Study design:

A prospective, randomized clinical trial was conducted to compare the efficacy of platelet-rich fibrin (PRF) and chitosan-based dressings in postoperative healing following mandibular third molar extractions. The study was approved by the institutional ethics committee and written informed consent was obtained from all participants.

## Participants:

A total of 60 patients aged 18-35 years, requiring bilateral mandibular third molar extractions, were included in the study. Patients with systemic conditions affecting healing (*e.g.*, diabetes, immunosuppression), smokers and those with active infections at the surgical site were excluded.

## Randomization and allocation:

Each patient served as their own control. The extraction sites were randomly allocated to receive either PRF or a chitosan-based dressing using a computer-generated randomization sequence.

## Surgical procedure:

The extractions were performed under local anesthesia using standard surgical techniques. After the removal of the third molars, hemostasis was achieved and the allocated dressing material was applied:

[1] PRF Group: PRF membranes were prepared by centrifuging the patient's blood at 2700 rpm for 12 minutes. The resultant fibrin clot was compressed into membranes and placed at the extraction site.

[2] Chitosan Group: Sterile chitosan-based dressings were placed directly into the extraction socket.

The wounds were sutured with 3-0 silk sutures in both groups. Postoperative instructions and medications (analgesics and antibiotics) were standardized for all patients.

## Outcome measures:

Postoperative evaluations were conducted on days 3, 7 and 14 by a blinded investigator. The following parameters were assessed:

[1] Pain: Measured using the Visual Analog Scale (VAS), ranging from 0 (no pain) to 10 (severe pain).

[2] Swelling: Assessed using facial measurements taken from specific anatomical landmarks.

[3] Wound Healing: Evaluated using Landry's Wound Healing Scale, which scores healing on a scale of 1 (poor) to 10 (excellent).

## Statistical analysis:

The data were analyzed using SPSS software (version 23). Paired t-tests were used to compare outcomes between the two groups and repeated measures ANOVA assessed changes over time. Statistical significance was set at p < 0.05.

## Results:

The PRF group showed significantly lower pain scores compared to the chitosan group at all postoperative time points (p < 0.05). Facial swelling was consistently reduced in the PRF group, with a highly significant difference observed on days 7 and 14 (p < 0.01). Wound healing scores were higher in the PRF group across all time points, indicating superior healing outcomes (p < 0.05) (Table 1, Table 2, Table 3). These findings suggest that PRF outperforms chitosan-based dressings in managing postoperative pain, reducing swelling and enhancing wound healing after mandibular third molar extractions.

## Discussion:

The findings of this study demonstrate that platelet-rich fibrin (PRF) is more effective than chitosan-based dressings in promoting postoperative healing following mandibular third molar extractions. PRF consistently outperformed chitosan in reducing pain, minimizing swelling and enhancing wound healing. These outcomes can be attributed to the unique biological and regenerative properties of PRF, which were not fully matched by the structural and antimicrobial benefits of chitosan-based dressings. Pain is one of the most critical parameters in assessing postoperative recovery as it directly impacts patient comfort and quality of life. In this study, patients treated with PRF reported consistently lower pain scores at all postoperative time points compared to those treated with chitosan-based dressings. This aligns with previous research, where PRF has been shown to possess strong anti-inflammatory properties, mediated through its ability to modulate the release of pro-inflammatory cytokines such as interleukin-6 (IL-6) and tumor necrosis factor-alpha (TNF-α) (1, 2). The fibrin matrix of PRF not only provides a protective barrier but also releases growth factors such as transforming growth factor-beta (TGF-β), which is known to play a role in reducing inflammation and promoting tissue regeneration [3]. In comparison, while chitosan dressings have been shown to alleviate pain to some extent, their mechanism is primarily indirect, relying on their antimicrobial action to reduce infection-associated inflammation [4, 5]. Unlike PRF, chitosan does not have direct anti-inflammatory effects, which may explain the less pronounced pain reduction observed in the chitosan group. This difference highlights the importance of biological signaling molecules, such as those present in PRF, in managing postoperative pain more effectively. Swelling is a common postoperative complication that results from the inflammatory response to tissue trauma during surgery. PRF significantly reduced postoperative swelling, a result attributed to its ability to promote angiogenesis and stabilize vascular integrity. The presence of vascular endothelial growth factor (VEGF) and platelet-derived growth factor (PDGF) in PRF enhances the formation of new blood vessels, improves oxygenation at the wound site and reduces edema [6]. Plasma rich in growth factors (PRGF-Endoret) is a natural, patient-derived therapy increasingly recognized in regenerative medicine for its ability to enhance and speed up tissue healing and bone regeneration [7].

Platelet-rich preparations are an emerging biotechnology designed to enhance tissue healing and bone regeneration. Their versatility and biocompatibility have led to widespread therapeutic applications across various medical and scientific disciplines, including dentistry, oral implantology, orthopedics, ulcer management, and tissue engineering [8]. While these properties are beneficial, they are not as effective in controlling postoperative swelling compared to the biologically active PRF. Wound healing scores further validated the superior efficacy of PRF in this study. PRF's fibrin network acts as a scaffold for cellular migration and proliferation, enabling fibroblasts and keratinocytes to rapidly colonize the wound site. Additionally, the sustained release of growth factors like TGF-β and PDGF from PRF stimulates collagen synthesis and epithelialization, critical for wound closure and tissue regeneration [9, 10]. This multifaceted biological activity ensures faster and more effective healing compared to chitosan-based dressings, which rely mainly on structural support and infection control. While chitosan is a proven wound dressing material with excellent biocompatibility and antimicrobial properties, it lacks the biological signaling molecules present in PRF. Its healing mechanism is primarily through maintaining a moist wound environment and preventing microbial contamination, which supports healing but does not actively accelerate tissue regeneration [1].

Despite these promising results, this study is not without limitations. The sample size of 60 patients, though adequate for preliminary findings, may limit the generalizability of the results. A larger cohort with diverse demographics and clinical conditions would provide more robust data. Furthermore, the follow-up period was limited to 14 days, which primarily captures soft tissue healing. Studies with extended follow-ups are needed to assess the long-term effects of PRF and chitosan on bone healing, scar formation and overall tissue regeneration. Cost and preparation time are additional considerations, particularly for PRF, which requires specific equipment and expertise for preparation. While it's superior efficacy may justify the investment, these factors could pose challenges in resource-limited clinical settings. Chitosan-based dressings, on the other hand, are more affordable and easier to use, making them a viable alternative when PRF is not available. Future research could explore the potential of combining PRF and chitosan-based dressings to leverage the biological benefits of PRF with the structural and antimicrobial properties of chitosan. Such a hybrid approach might enhance healing outcomes further while addressing cost and practicality concerns. Additionally, studies investigating the molecular mechanisms underlying the effects of these materials could provide deeper insights into their roles in wound healing.

## Conclusion:

Platelet-rich fibrin showed superior efficacy over chitosan-based dressings in managing postoperative healing after mandibular third molar extractions. Its biological properties, including anti-inflammatory effects with growth factor release make it an ideal choice for enhancing recovery.

## Figures and Tables

**Table 1:** Comparison of postoperative pain (VAS Scores) between PRF and chitosan groups

**Day**	**PRF Group (Mean ± SD)**	**Chitosan Group (Mean ± SD)**	**p-value**
3	3.5 ± 0.8	4.2 ± 1.1	0.03*
7	2.0 ± 0.6	2.8 ± 0.9	0.02*
14	0.5 ± 0.3	1.2 ± 0.5	0.01*
*Significant difference between groups (p < 0.05).

**Table 2:** Comparison of facial swelling (Measured in mm) between PRF and chitosan groups

**Day**	**PRF Group (Mean ± SD)**	**Chitosan Group (Mean ± SD)**	**p-value**
3	12.5 ± 2.3	15.8 ± 2.9	0.01*
7	5.8 ± 1.7	9.2 ± 2.1	0.001**
14	1.2 ± 0.6	3.5 ± 0.8	0.001**
*Significant difference (p < 0.05).
**Highly significant difference (p < 0.01).

**Table 3:** Comparison of wound healing scores (Landry's Scale) between PRF and chitosan groups

**Day**	**PRF Group (Mean ± SD)**	**Chitosan Group (Mean ± SD)**	**p-value**
3	5.6 ± 0.9	4.8 ± 0.7	0.04*
7	8.5 ± 0.8	7.6 ± 0.9	0.02*
14	9.2 ± 0.6	8.1 ± 0.9	0.01*
*Significant difference between groups (p < 0.05).
